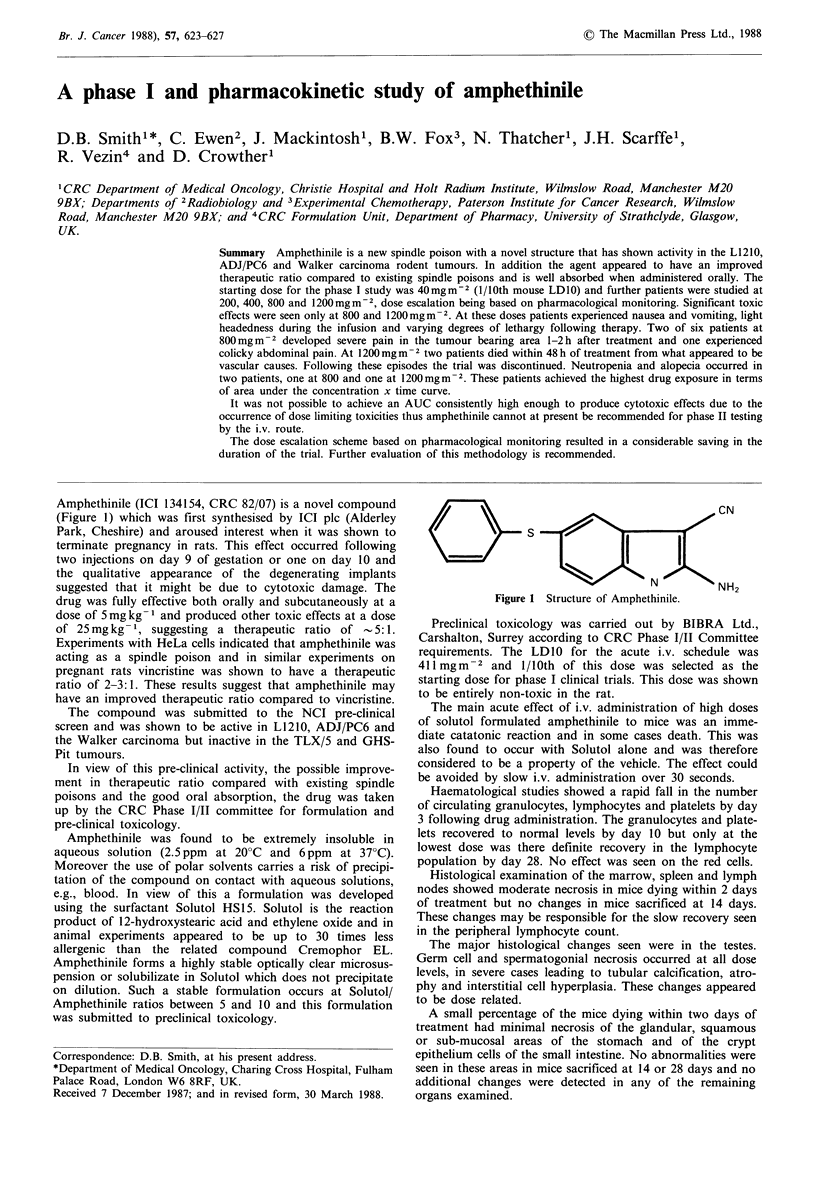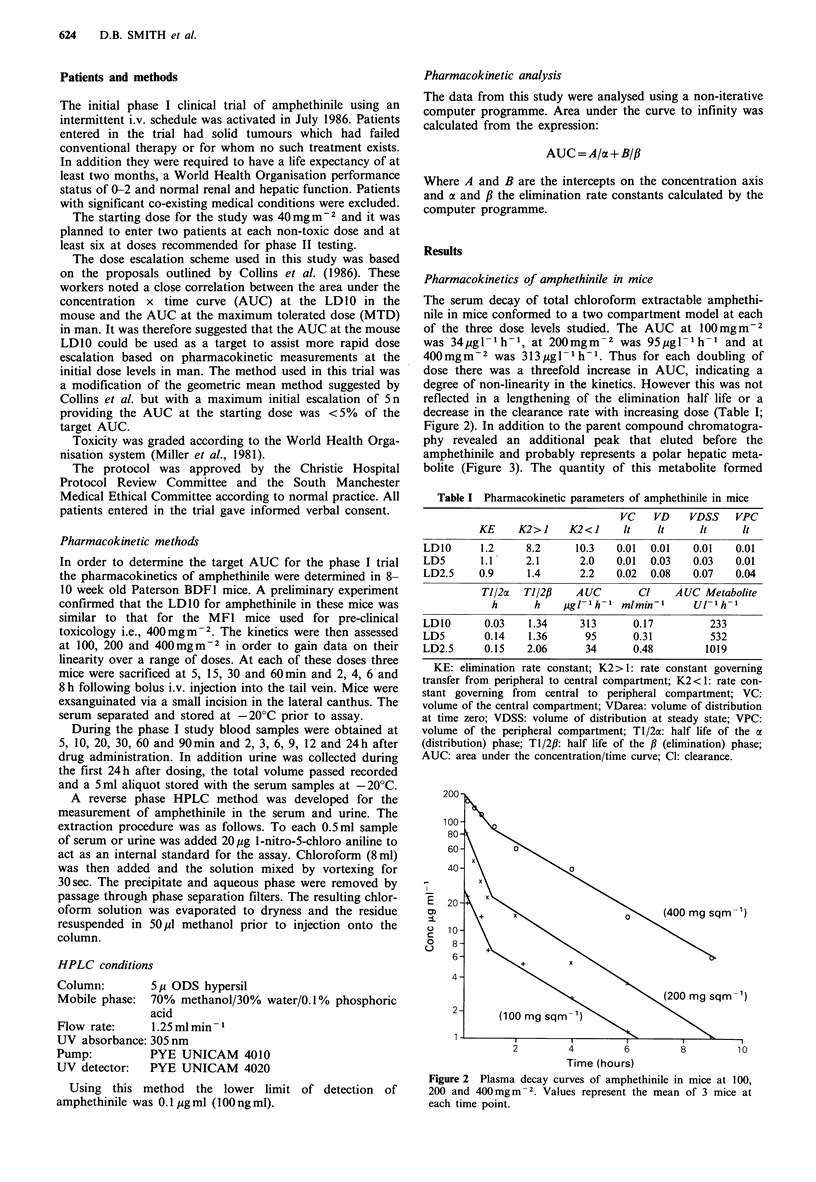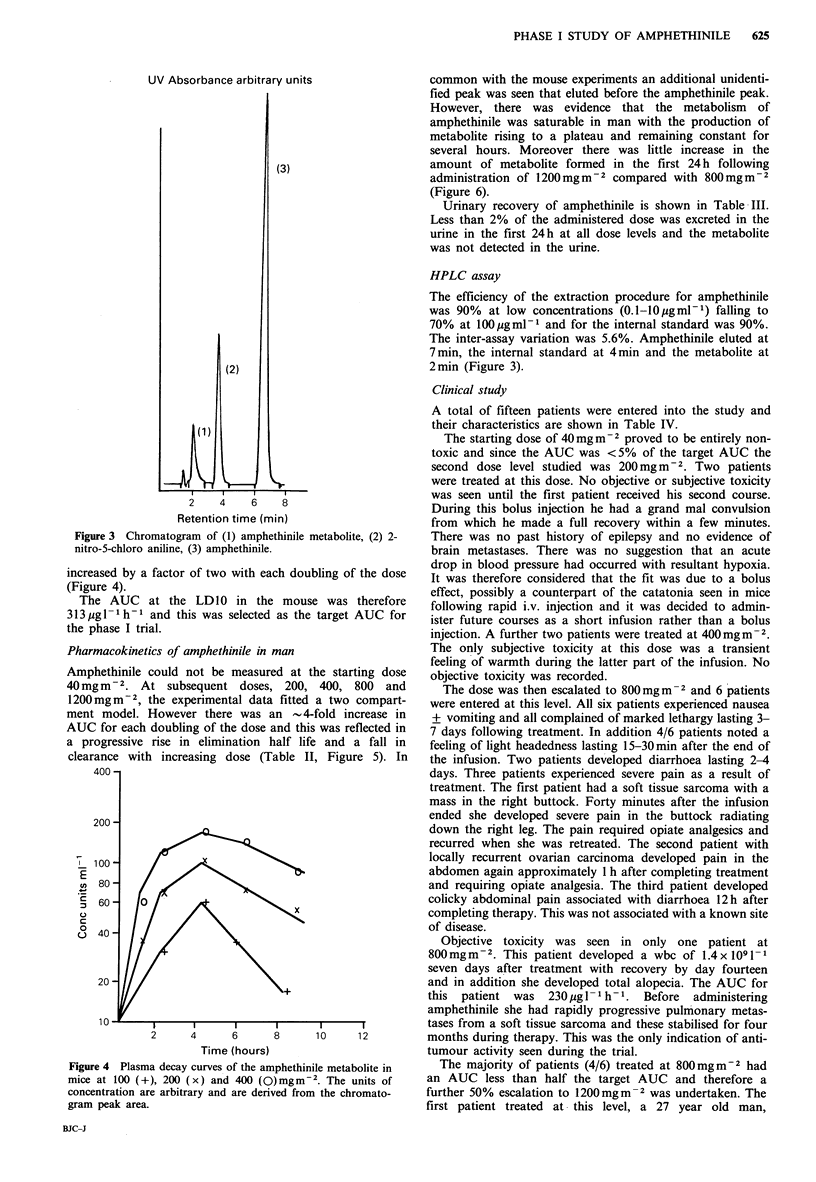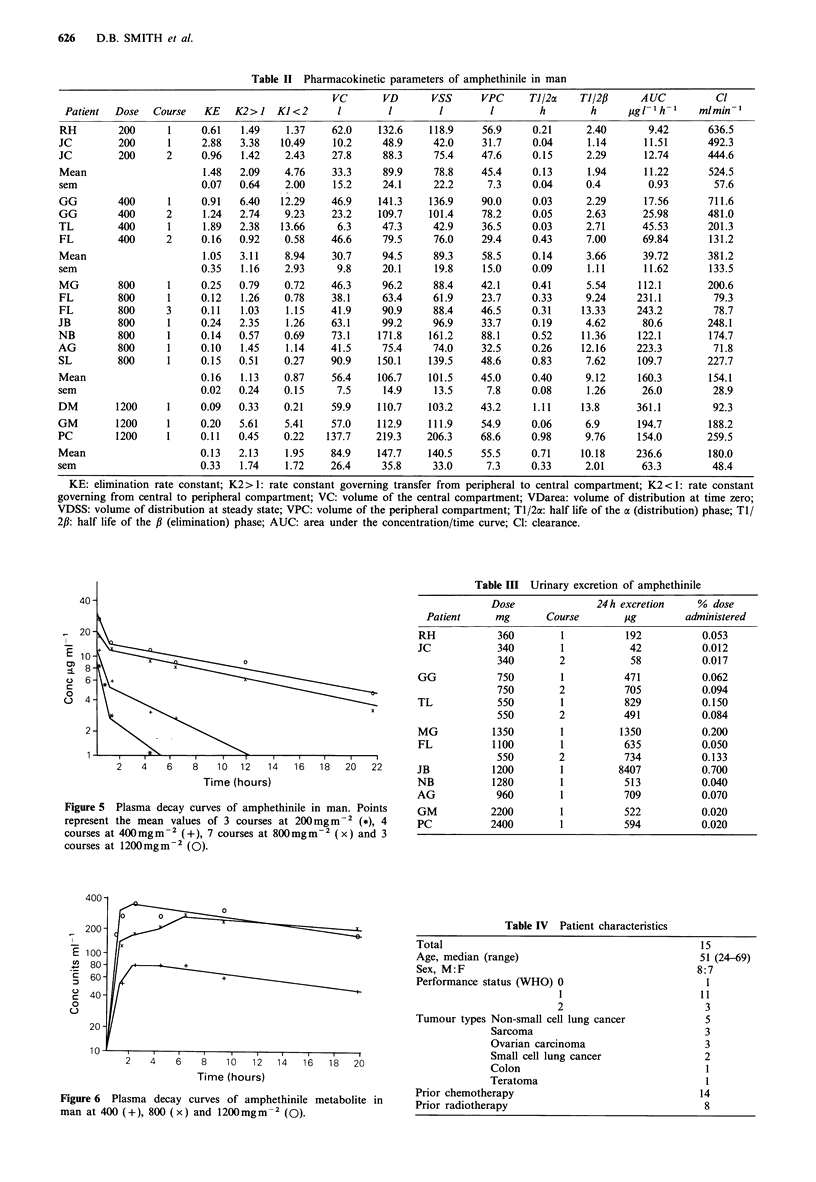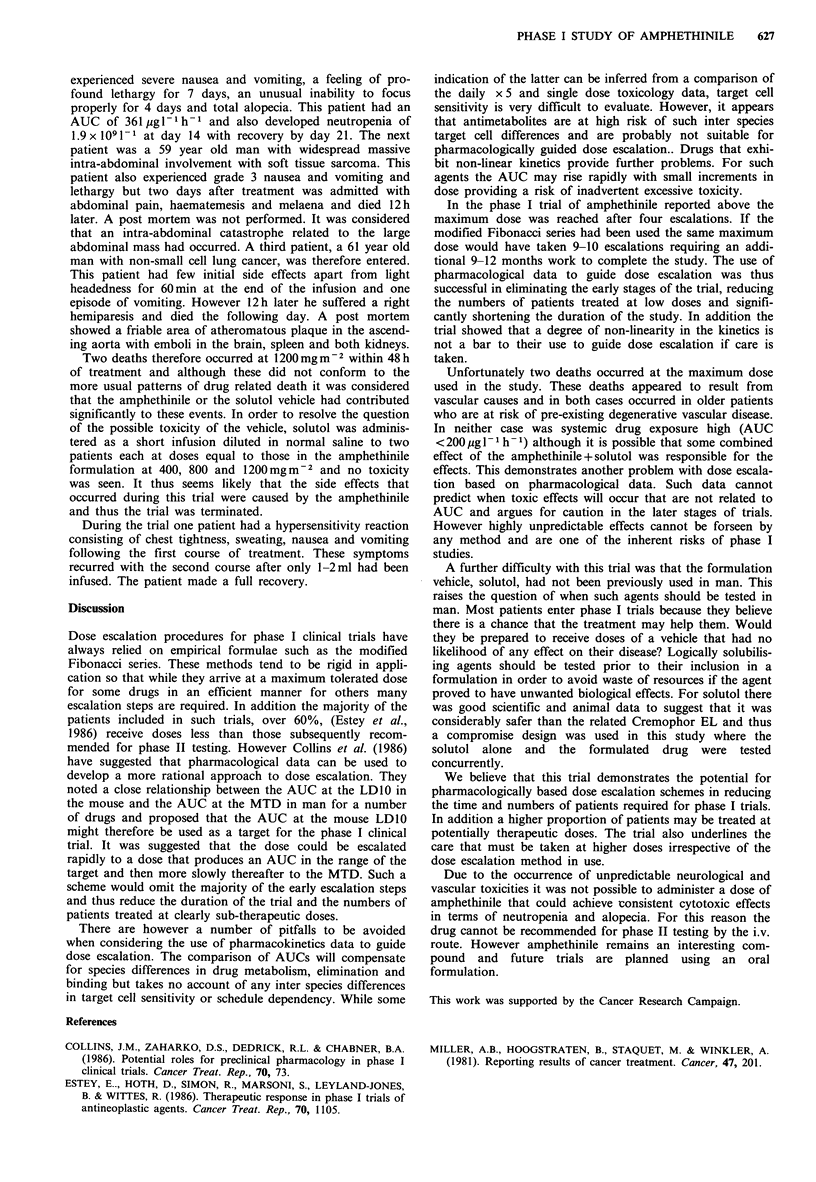# A phase I and pharmacokinetic study of amphethinile.

**DOI:** 10.1038/bjc.1988.142

**Published:** 1988-06

**Authors:** D. B. Smith, C. Ewen, J. Mackintosh, B. W. Fox, N. Thatcher, J. H. Scarffe, R. Vezin, D. Crowther

**Affiliations:** CRC Department of Medical Oncology, Christie Hospital, Manchester, UK.

## Abstract

Amphethinile is a new spindle poison with a novel structure that has shown activity in the L1210, ADJ/PC6 and Walker carcinoma rodent tumours. In addition the agent appeared to have an improved therapeutic ratio compared to existing spindle poisons and is well absorbed when administered orally. The starting dose for the phase I study was 40 mg m-2 (1/10th mouse LD10) and further patients were studied at 200, 400, 800 and 1200 mg m-2, dose escalation being based on pharmacological monitoring. Significant toxic effects were seen only at 800 and 1200 mg m-2. At these doses patients experienced nausea and vomiting, light headedness during the infusion and varying degrees of lethargy following therapy. Two of six patients at 800 mg m-2 developed severe pain in the tumour bearing area 1-2 h after treatment and one experienced colicky abdominal pain. At 1200 mg m-2 two patients died within 48 h of treatment from what appeared to be vascular causes. Following these episodes the trial was discontinued. Neutropenia and alopecia occurred in two patients, one at 800 and one at 1200 mg m-2. These patients achieved the highest drug exposure in terms of area under the concentration x time curve. It was not possible to achieve an AUC consistently high enough to produce cytotoxic effects due to the occurrence of dose limiting toxicities thus amphethinile cannot at present be recommended for phase II testing by the i.v. route. The dose escalation scheme based on pharmacological monitoring resulted in a considerable saving in the duration of the trial. Further evaluation of this methodology is recommended.


Br. J. Cancer 1988), 57, 623-627

© The Macmillan Press Ltd., 1988

### A phase I and pharmacokinetic study of amphethinile

D.B. Smith[1]*, C. Ewen[2], J. Mackintosh[1], B.W. Fox[3], N. Thatcher[1], J.H. Scarffe[1], R. Vezin[4] and D. Crowther[1]

[1]*CRC Department of Medical Oncology, Christie Hospital and Holt Radium Institute, Wilmslow Road, Manchester M20 9BX; Departments of* [2]*Radiobiology and* [3]*Experimental Chemotherapy, Paterson Institute for Cancer Research, Wilmslow Road, Manchester M20 9BX; and* [4]*CRC Formulation Unit, Department of Pharmacy, University of Strathclyde, Glasgow, UK.*

**Summary** Amphethinile is a new spindle poison with a novel structure that has shown activity in the L1210, ADJ/PC6 and Walker carcinoma rodent tumours. In addition the agent appeared to have an improved therapeutic ratio compared to existing spindle poisons and is well absorbed when administered orally. The starting dose for the phase I study was $40\,mg\,m^{-2}$ (1/10th mouse LD10) and further patients were studied at 200, 400, 800 and $1200\,mg\,m^{-2}$, dose escalation being based on pharmacological monitoring. Significant toxic effects were seen only at 800 and $1200\,mg\,m^{-2}$. At these doses patients experienced nausea and vomiting, light headedness during the infusion and varying degrees of lethargy following therapy. Two of six patients at $800\,mg\,m^{-2}$ developed severe pain in the tumour bearing area 1–2h after treatment and one experienced colicky abdominal pain. At $1200\,mg\,m^{-2}$ two patients died within 48h of treatment from what appeared to be vascular causes. Following these episodes the trial was discontinued. Neutropenia and alopecia occurred in two patients, one at 800 and one at $1200\,mg\,m^{-2}$. These patients achieved the highest drug exposure in terms of area under the concentration $x$ time curve.

It was not possible to achieve an AUC consistently high enough to produce cytotoxic effects due to the occurrence of dose limiting toxicities thus amphethinile cannot at present be recommended for phase II testing by the i.v. route.

The dose escalation scheme based on pharmacological monitoring resulted in a considerable saving in the duration of the trial. Further evaluation of this methodology is recommended.

Amphethinile (ICI 134154, CRC 82/07) is a novel compound (Figure 1) which was first synthesised by ICI plc (Alderley Park, Cheshire) and aroused interest when it was shown to terminate pregnancy in rats. This effect occurred following two injections on day 9 of gestation or one on day 10 and the qualitative appearance of the degenerating implants suggested that it might be due to cytotoxic damage. The drug was fully effective both orally and subcutaneously at a dose of $5\,mg\,kg^{-1}$ and produced other toxic effects at a dose of $25\,mg\,kg^{-1}$, suggesting a therapeutic ratio of $\sim 5:1$. Experiments with HeLa cells indicated that amphethinile was acting as a spindle poison and in similar experiments on pregnant rats vincristine was shown to have a therapeutic ratio of 2–3:1. These results suggest that amphethinile may have an improved therapeutic ratio compared to vincristine.

The compound was submitted to the NCI pre-clinical screen and was shown to be active in L1210, ADJ/PC6 and the Walker carcinoma but inactive in the TLX/5 and GHS-Pit tumours.

In view of this pre-clinical activity, the possible improvement in therapeutic ratio compared with existing spindle poisons and the good oral absorption, the drug was taken up by the CRC Phase I/II committee for formulation and pre-clinical toxicology.

Amphethinile was found to be extremely insoluble in aqueous solution (2.5ppm at 20°C and 6ppm at 37°C). Moreover the use of polar solvents carries a risk of precipitation of the compound on contact with aqueous solutions, e.g., blood. In view of this a formulation was developed using the surfactant Solutol HS15. Solutol is the reaction product of 12-hydroxystearic acid and ethylene oxide and in animal experiments appeared to be up to 30 times less allergenic than the related compound Cremophor EL. Amphethinile forms a highly stable optically clear microsuspension or solubilizate in Solutol which does not precipitate on dilution. Such a stable formulation occurs at Solutol/Amphethinile ratios between 5 and 10 and this formulation was submitted to preclinical toxicology.

**Figure 1** Structure of Amphethinile.

Preclinical toxicology was carried out by BIBRA Ltd., Carshalton, Surrey according to CRC Phase I/II Committee requirements. The LD10 for the acute i.v. schedule was $411\,mg\,m^{-2}$ and 1/10th of this dose was selected as the starting dose for phase I clinical trials. This dose was shown to be entirely non-toxic in the rat.

The main acute effect of i.v. administration of high doses of solutol formulated amphethinile to mice was an immediate catatonic reaction and in some cases death. This was also found to occur with Solutol alone and was therefore considered to be a property of the vehicle. The effect could be avoided by slow i.v. administration over 30 seconds.

Haematological studies showed a rapid fall in the number of circulating granulocytes, lymphocytes and platelets by day 3 following drug administration. The granulocytes and platelets recovered to normal levels by day 10 but only at the lowest dose was there definite recovery in the lymphocyte population by day 28. No effect was seen on the red cells.

Histological examination of the marrow, spleen and lymph nodes showed moderate necrosis in mice dying within 2 days of treatment but no changes in mice sacrificed at 14 days. These changes may be responsible for the slow recovery seen in the peripheral lymphocyte count.

The major histological changes seen were in the testes. Germ cell and spermatogonial necrosis occurred at all dose levels, in severe cases leading to tubular calcification, atrophy and interstitial cell hyperplasia. These changes appeared to be dose related.

A small percentage of the mice dying within two days of treatment had minimal necrosis of the glandular, squamous or sub-mucosal areas of the stomach and of the crypt epithelium cells of the small intestine. No abnormalities were seen in these areas in mice sacrificed at 14 or 28 days and no additional changes were detected in any of the remaining organs examined.

Correspondence: D.B. Smith, at his present address.
*Department of Medical Oncology, Charing Cross Hospital, Fulham Palace Road, London W6 8RF, UK.
Received 7 December 1987; and in revised form, 30 March 1988.

### Patients and methods

The initial phase I clinical trial of amphethinile using an intermittent i.v. schedule was activated in July 1986. Patients entered in the trial had solid tumours which had failed conventional therapy or for whom no such treatment exists. In addition they were required to have a life expectancy of at least two months, a World Health Organisation performance status of 0–2 and normal renal and hepatic function. Patients with significant co-existing medical conditions were excluded.

The starting dose for the study was $40\,mg\,m^{-2}$ and it was planned to enter two patients at each non-toxic dose and at least six at doses recommended for phase II testing.

The dose escalation scheme used in this study was based on the proposals outlined by Collins *et al.* (1986). These workers noted a close correlation between the area under the concentration × time curve (AUC) at the LD10 in the mouse and the AUC at the maximum tolerated dose (MTD) in man. It was therefore suggested that the AUC at the mouse LD10 could be used as a target to assist more rapid dose escalation based on pharmacokinetic measurements at the initial dose levels in man. The method used in this trial was a modification of the geometric mean method suggested by Collins *et al.* but with a maximum initial escalation of 5n providing the AUC at the starting dose was <5% of the target AUC.

Toxicity was graded according to the World Health Organisation system (Miller *et al.*, 1981).

The protocol was approved by the Christie Hospital Protocol Review Committee and the South Manchester Medical Ethical Committee according to normal practice. All patients entered in the trial gave informed verbal consent.

### *Pharmacokinetic methods*

In order to determine the target AUC for the phase I trial the pharmacokinetics of amphethinile were determined in 8–10 week old Paterson BDF1 mice. A preliminary experiment confirmed that the LD10 for amphethinile in these mice was similar to that for the MF1 mice used for pre-clinical toxicology i.e., $400\,mg\,m^{-2}$. The kinetics were then assessed at 100, 200 and $400\,mg\,m^{-2}$ in order to gain data on their linearity over a range of doses. At each of these doses three mice were sacrificed at 5, 15, 30 and 60 min and 2, 4, 6 and 8 h following bolus i.v. injection into the tail vein. Mice were exsanguinated via a small incision in the lateral canthus. The serum separated and stored at −20°C prior to assay.

During the phase I study blood samples were obtained at 5, 10, 20, 30, 60 and 90 min and 2, 3, 6, 9, 12 and 24 h after drug administration. In addition urine was collected during the first 24 h after dosing, the total volume passed recorded and a 5 ml aliquot stored with the serum samples at −20°C.

A reverse phase HPLC method was developed for the measurement of amphethinile in the serum and urine. The extraction procedure was as follows. To each 0.5 ml sample of serum or urine was added 20 μg 1-nitro-5-chloro aniline to act as an internal standard for the assay. Chloroform (8 ml) was then added and the solution mixed by vortexing for 30 sec. The precipitate and aqueous phase were removed by passage through phase separation filters. The resulting chloroform solution was evaporated to dryness and the residue resuspended in 50 μl methanol prior to injection onto the column.

### *HPLC conditions*

Column: 5 μ ODS hypersil
Mobile phase: 70% methanol/30% water/0.1% phosphoric acid
Flow rate: $1.25\,ml\,min^{-1}$
UV absorbance: 305 nm
Pump: PYE UNICAM 4010
UV detector: PYE UNICAM 4020

Using this method the lower limit of detection of amphethinile was 0.1 μg ml (100 ng ml).

### *Pharmacokinetic analysis*

The data from this study were analysed using a non-iterative computer programme. Area under the curve to infinity was calculated from the expression:

$$AUC = A/\alpha + B/\beta$$

Where $A$ and $B$ are the intercepts on the concentration axis and $\alpha$ and $\beta$ the elimination rate constants calculated by the computer programme.

### Results

### *Pharmacokinetics of amphethinile in mice*

The serum decay of total chloroform extractable amphethinile in mice conformed to a two compartment model at each of the three dose levels studied. The AUC at $100\,mg\,m^{-2}$ was $34\,\mu g\,l^{-1}\,h^{-1}$, at $200\,mg\,m^{-2}$ was $95\,\mu g\,l^{-1}\,h^{-1}$ and at $400\,mg\,m^{-2}$ was $313\,\mu g\,l^{-1}\,h^{-1}$. Thus for each doubling of dose there was a threefold increase in AUC, indicating a degree of non-linearity in the kinetics. However this was not reflected in a lengthening of the elimination half life or a decrease in the clearance rate with increasing dose (Table I; Figure 2). In addition to the parent compound chromatography revealed an additional peak that eluted before the amphethinile and probably represents a polar hepatic metabolite (Figure 3). The quantity of this metabolite formed

**Table I** Pharmacokinetic parameters of amphethinile in mice

| | *KE* | *K2>1* | *K2<1* | *VC lt* | *VD lt* | *VDSS lt* | *VPC lt* |
|---|---|---|---|---|---|---|---|
| LD10 | 1.2 | 8.2 | 10.3 | 0.01 | 0.01 | 0.01 | 0.01 |
| LD5 | 1.1 | 2.1 | 2.0 | 0.01 | 0.03 | 0.03 | 0.01 |
| LD2.5 | 0.9 | 1.4 | 2.2 | 0.02 | 0.08 | 0.07 | 0.04 |

| | *T1/2α h* | *T1/2β h* | *AUC* $\mu g\,l^{-1}\,h^{-1}$ | *Cl* $ml\,min^{-1}$ | *AUC Metabolite* $U\,l^{-1}\,h^{-1}$ |
|---|---|---|---|---|---|
| LD10 | 0.03 | 1.34 | 313 | 0.17 | 233 |
| LD5 | 0.14 | 1.36 | 95 | 0.31 | 532 |
| LD2.5 | 0.15 | 2.06 | 34 | 0.48 | 1019 |

KE: elimination rate constant; K2>1: rate constant governing transfer from peripheral to central compartment; K2<1: rate constant governing from central to peripheral compartment; VC: volume of the central compartment; VDarea: volume of distribution at time zero; VDSS: volume of distribution at steady state; VPC: volume of the peripheral compartment; T1/2α: half life of the α (distribution) phase; T1/2β: half life of the β (elimination) phase; AUC: area under the concentration/time curve; Cl: clearance.

**Figure 2** Plasma decay curves of amphethinile in mice at 100, 200 and $400\,mg\,m^{-2}$. Values represent the mean of 3 mice at each time point.

**Figure 3** Chromatogram of (1) amphethinile metabolite, (2) 2-nitro-5-chloro aniline, (3) amphethinile.

increased by a factor of two with each doubling of the dose (Figure 4).

The AUC at the LD10 in the mouse was therefore $313\,\mu g\,l^{-1}\,h^{-1}$ and this was selected as the target AUC for the phase I trial.

*Pharmacokinetics of amphethinile in man*

Amphethinile could not be measured at the starting dose $40\,mg\,m^{-2}$. At subsequent doses, 200, 400, 800 and $1200\,mg\,m^{-2}$, the experimental data fitted a two compartment model. However there was an ~4-fold increase in AUC for each doubling of the dose and this was reflected in a progressive rise in elimination half life and a fall in clearance with increasing dose (Table II, Figure 5). In common with the mouse experiments an additional unidentified peak was seen that eluted before the amphethinile peak. However, there was evidence that the metabolism of amphethinile was saturable in man with the production of metabolite rising to a plateau and remaining constant for several hours. Moreover there was little increase in the amount of metabolite formed in the first 24 h following administration of $1200\,mg\,m^{-2}$ compared with $800\,mg\,m^{-2}$ (Figure 6).

**Figure 4** Plasma decay curves of the amphethinile metabolite in mice at 100 (+), 200 (×) and 400 (○) $mg\,m^{-2}$. The units of concentration are arbitrary and are derived from the chromatogram peak area.

Urinary recovery of amphethinile is shown in Table III. Less than 2% of the administered dose was excreted in the urine in the first 24 h at all dose levels and the metabolite was not detected in the urine.

*HPLC assay*

The efficiency of the extraction procedure for amphethinile was 90% at low concentrations ($0.1$–$10\,\mu g\,ml^{-1}$) falling to 70% at $100\,\mu g\,ml^{-1}$ and for the internal standard was 90%. The inter-assay variation was 5.6%. Amphethinile eluted at 7 min, the internal standard at 4 min and the metabolite at 2 min (Figure 3).

*Clinical study*

A total of fifteen patients were entered into the study and their characteristics are shown in Table IV.

The starting dose of $40\,mg\,m^{-2}$ proved to be entirely non-toxic and since the AUC was <5% of the target AUC the second dose level studied was $200\,mg\,m^{-2}$. Two patients were treated at this dose. No objective or subjective toxicity was seen until the first patient received his second course. During this bolus injection he had a grand mal convulsion from which he made a full recovery within a few minutes. There was no past history of epilepsy and no evidence of brain metastases. There was no suggestion that an acute drop in blood pressure had occurred with resultant hypoxia. It was therefore considered that the fit was due to a bolus effect, possibly a counterpart of the catatonia seen in mice following rapid i.v. injection and it was decided to administer future courses as a short infusion rather than a bolus injection. A further two patients were treated at $400\,mg\,m^{-2}$. The only subjective toxicity at this dose was a transient feeling of warmth during the latter part of the infusion. No objective toxicity was recorded.

The dose was then escalated to $800\,mg\,m^{-2}$ and 6 patients were entered at this level. All six patients experienced nausea ± vomiting and all complained of marked lethargy lasting 3–7 days following treatment. In addition 4/6 patients noted a feeling of light headedness lasting 15–30 min after the end of the infusion. Two patients developed diarrhoea lasting 2–4 days. Three patients experienced severe pain as a result of treatment. The first patient had a soft tissue sarcoma with a mass in the right buttock. Forty minutes after the infusion ended she developed severe pain in the buttock radiating down the right leg. The pain required opiate analgesics and recurred when she was retreated. The second patient with locally recurrent ovarian carcinoma developed pain in the abdomen again approximately 1 h after completing treatment and requiring opiate analgesia. The third patient developed colicky abdominal pain associated with diarrhoea 12 h after completing therapy. This was not associated with a known site of disease.

Objective toxicity was seen in only one patient at $800\,mg\,m^{-2}$. This patient developed a wbc of $1.4\times10^{9}\,l^{-1}$ seven days after treatment with recovery by day fourteen and in addition she developed total alopecia. The AUC for this patient was $230\,\mu g\,l^{-1}\,h^{-1}$. Before administering amphethinile she had rapidly progressive pulmonary metastases from a soft tissue sarcoma and these stabilised for four months during therapy. This was the only indication of anti-tumour activity seen during the trial.

The majority of patients (4/6) treated at $800\,mg\,m^{-2}$ had an AUC less than half the target AUC and therefore a further 50% escalation to $1200\,mg\,m^{-2}$ was undertaken. The first patient treated at this level, a 27 year old man,

**Table II** Pharmacokinetic parameters of amphethinile in man

| *Patient* | *Dose* | *Course* | *KE* | *K2>1* | *K1<2* | *VC* *l* | *VD* *l* | *VSS* *l* | *VPC* *l* | *T1/2α* *h* | *T1/2β* *h* | *AUC* $\mu g\,l^{-1}\,h^{-1}$ | *Cl* $ml\,min^{-1}$ |
|---|---|---|---|---|---|---|---|---|---|---|---|---|---|
| RH | 200 | 1 | 0.61 | 1.49 | 1.37 | 62.0 | 132.6 | 118.9 | 56.9 | 0.21 | 2.40 | 9.42 | 636.5 |
| JC | 200 | 1 | 2.88 | 3.38 | 10.49 | 10.2 | 48.9 | 42.0 | 31.7 | 0.04 | 1.14 | 11.51 | 492.3 |
| JC | 200 | 2 | 0.96 | 1.42 | 2.43 | 27.8 | 88.3 | 75.4 | 47.6 | 0.15 | 2.29 | 12.74 | 444.6 |
| Mean | | | 1.48 | 2.09 | 4.76 | 33.3 | 89.9 | 78.8 | 45.4 | 0.13 | 1.94 | 11.22 | 524.5 |
| sem | | | 0.07 | 0.64 | 2.00 | 15.2 | 24.1 | 22.2 | 7.3 | 0.04 | 0.4 | 0.93 | 57.6 |
| GG | 400 | 1 | 0.91 | 6.40 | 12.29 | 46.9 | 141.3 | 136.9 | 90.0 | 0.03 | 2.29 | 17.56 | 711.6 |
| GG | 400 | 2 | 1.24 | 2.74 | 9.23 | 23.2 | 109.7 | 101.4 | 78.2 | 0.05 | 2.63 | 25.98 | 481.0 |
| TL | 400 | 1 | 1.89 | 2.38 | 13.66 | 6.3 | 47.3 | 42.9 | 36.5 | 0.03 | 2.71 | 45.53 | 201.3 |
| FL | 400 | 2 | 0.16 | 0.92 | 0.58 | 46.6 | 79.5 | 76.0 | 29.4 | 0.43 | 7.00 | 69.84 | 131.2 |
| Mean | | | 1.05 | 3.11 | 8.94 | 30.7 | 94.5 | 89.3 | 58.5 | 0.14 | 3.66 | 39.72 | 381.2 |
| sem | | | 0.35 | 1.16 | 2.93 | 9.8 | 20.1 | 19.8 | 15.0 | 0.09 | 1.11 | 11.62 | 133.5 |
| MG | 800 | 1 | 0.25 | 0.79 | 0.72 | 46.3 | 96.2 | 88.4 | 42.1 | 0.41 | 5.54 | 112.1 | 200.6 |
| FL | 800 | 1 | 0.12 | 1.26 | 0.78 | 38.1 | 63.4 | 61.9 | 23.7 | 0.33 | 9.24 | 231.1 | 79.3 |
| FL | 800 | 3 | 0.11 | 1.03 | 1.15 | 41.9 | 90.9 | 88.4 | 46.5 | 0.31 | 13.33 | 243.2 | 78.7 |
| JB | 800 | 1 | 0.24 | 2.35 | 1.26 | 63.1 | 99.2 | 96.9 | 33.7 | 0.19 | 4.62 | 80.6 | 248.1 |
| NB | 800 | 1 | 0.14 | 0.57 | 0.69 | 73.1 | 171.8 | 161.2 | 88.1 | 0.52 | 11.36 | 122.1 | 174.7 |
| AG | 800 | 1 | 0.10 | 1.45 | 1.14 | 41.5 | 75.4 | 74.0 | 32.5 | 0.26 | 12.16 | 223.3 | 71.8 |
| SL | 800 | 1 | 0.15 | 0.51 | 0.27 | 90.9 | 150.1 | 139.5 | 48.6 | 0.83 | 7.62 | 109.7 | 227.7 |
| Mean | | | 0.16 | 1.13 | 0.87 | 56.4 | 106.7 | 101.5 | 45.0 | 0.40 | 9.12 | 160.3 | 154.1 |
| sem | | | 0.02 | 0.24 | 0.15 | 7.5 | 14.9 | 13.5 | 7.8 | 0.08 | 1.26 | 26.0 | 28.9 |
| DM | 1200 | 1 | 0.09 | 0.33 | 0.21 | 59.9 | 110.7 | 103.2 | 43.2 | 1.11 | 13.8 | 361.1 | 92.3 |
| GM | 1200 | 1 | 0.20 | 5.61 | 5.41 | 57.0 | 112.9 | 111.9 | 54.9 | 0.06 | 6.9 | 194.7 | 188.2 |
| PC | 1200 | 1 | 0.11 | 0.45 | 0.22 | 137.7 | 219.3 | 206.3 | 68.6 | 0.98 | 9.76 | 154.0 | 259.5 |
| Mean | | | 0.13 | 2.13 | 1.95 | 84.9 | 147.7 | 140.5 | 55.5 | 0.71 | 10.18 | 236.6 | 180.0 |
| sem | | | 0.33 | 1.74 | 1.72 | 26.4 | 35.8 | 33.0 | 7.3 | 0.33 | 2.01 | 63.3 | 48.4 |

KE: elimination rate constant; K2>1: rate constant governing transfer from peripheral to central compartment; K2<1: rate constant governing from central to peripheral compartment; VC: volume of the central compartment; VDarea: volume of distribution at time zero; VDSS: volume of distribution at steady state; VPC: volume of the peripheral compartment; T1/2α: half life of the α (distribution) phase; T1/2β: half life of the β (elimination) phase; AUC: area under the concentration/time curve; Cl: clearance.

**Figure 5** Plasma decay curves of amphethinile in man. Points represent the mean values of 3 courses at $200\,mg\,m^{-2}$ (*), 4 courses at $400\,mg\,m^{-2}$ (+), 7 courses at $800\,mg\,m^{-2}$ (×) and 3 courses at $1200\,mg\,m^{-2}$ (○).

**Table III** Urinary excretion of amphethinile

| *Patient* | *Dose* *mg* | *Course* | *24 h excretion* *μg* | *% dose administered* |
|---|---|---|---|---|
| RH | 360 | 1 | 192 | 0.053 |
| JC | 340 | 1 | 42 | 0.012 |
| | 340 | 2 | 58 | 0.017 |
| GG | 750 | 1 | 471 | 0.062 |
| | 750 | 2 | 705 | 0.094 |
| TL | 550 | 1 | 829 | 0.150 |
| | 550 | 2 | 491 | 0.084 |
| MG | 1350 | 1 | 1350 | 0.200 |
| FL | 1100 | 1 | 635 | 0.050 |
| | 550 | 2 | 734 | 0.133 |
| JB | 1200 | 1 | 8407 | 0.700 |
| NB | 1280 | 1 | 513 | 0.040 |
| AG | 960 | 1 | 709 | 0.070 |
| GM | 2200 | 1 | 522 | 0.020 |
| PC | 2400 | 1 | 594 | 0.020 |

**Figure 6** Plasma decay curves of amphethinile metabolite in man at 400 (+), 800 (×) and $1200\,mg\,m^{-2}$ (○).

**Table IV** Patient characteristics

| | |
|---|---|
| Total | 15 |
| Age, median (range) | 51 (24–69) |
| Sex, M:F | 8:7 |
| Performance status (WHO) 0 | 1 |
| 1 | 11 |
| 2 | 3 |
| Tumour types Non-small cell lung cancer | 5 |
| Sarcoma | 3 |
| Ovarian carcinoma | 3 |
| Small cell lung cancer | 2 |
| Colon | 1 |
| Teratoma | 1 |
| Prior chemotherapy | 14 |
| Prior radiotherapy | 8 |

experienced severe nausea and vomiting, a feeling of profound lethargy for 7 days, an unusual inability to focus properly for 4 days and total alopecia. This patient had an AUC of $361\,\mu g\,l^{-1}\,h^{-1}$ and also developed neutropenia of $1.9 \times 10^9\,l^{-1}$ at day 14 with recovery by day 21. The next patient was a 59 year old man with widespread massive intra-abdominal involvement with soft tissue sarcoma. This patient also experienced grade 3 nausea and vomiting and lethargy but two days after treatment was admitted with abdominal pain, haematemesis and melaena and died 12h later. A post mortem was not performed. It was considered that an intra-abdominal catastrophe related to the large abdominal mass had occurred. A third patient, a 61 year old man with non-small cell lung cancer, was therefore entered. This patient had few initial side effects apart from light headedness for 60min at the end of the infusion and one episode of vomiting. However 12h later he suffered a right hemiparesis and died the following day. A post mortem showed a friable area of atheromatous plaque in the ascending aorta with emboli in the brain, spleen and both kidneys.

Two deaths therefore occurred at $1200\,mg\,m^{-2}$ within 48h of treatment and although these did not conform to the more usual patterns of drug related death it was considered that the amphethinile or the solutol vehicle had contributed significantly to these events. In order to resolve the question of the possible toxicity of the vehicle, solutol was administered as a short infusion diluted in normal saline to two patients each at doses equal to those in the amphethinile formulation at 400, 800 and $1200\,mg\,m^{-2}$ and no toxicity was seen. It thus seems likely that the side effects that occurred during this trial were caused by the amphethinile and thus the trial was terminated.

During the trial one patient had a hypersensitivity reaction consisting of chest tightness, sweating, nausea and vomiting following the first course of treatment. These symptoms recurred with the second course after only 1–2ml had been infused. The patient made a full recovery.

### Discussion

Dose escalation procedures for phase I clinical trials have always relied on empirical formulae such as the modified Fibonacci series. These methods tend to be rigid in application so that while they arrive at a maximum tolerated dose for some drugs in an efficient manner for others many escalation steps are required. In addition the majority of the patients included in such trials, over 60%, (Estey *et al.*, 1986) receive doses less than those subsequently recommended for phase II testing. However Collins *et al.* (1986) have suggested that pharmacological data can be used to develop a more rational approach to dose escalation. They noted a close relationship between the AUC at the LD10 in the mouse and the AUC at the MTD in man for a number of drugs and proposed that the AUC at the mouse LD10 might therefore be used as a target for the phase I clinical trial. It was suggested that the dose could be escalated rapidly to a dose that produces an AUC in the range of the target and then more slowly thereafter to the MTD. Such a scheme would omit the majority of the early escalation steps and thus reduce the duration of the trial and the numbers of patients treated at clearly sub-therapeutic doses.

There are however a number of pitfalls to be avoided when considering the use of pharmacokinetics data to guide dose escalation. The comparison of AUCs will compensate for species differences in drug metabolism, elimination and binding but takes no account of any inter species differences in target cell sensitivity or schedule dependency. While some indication of the latter can be inferred from a comparison of the daily $\times 5$ and single dose toxicology data, target cell sensitivity is very difficult to evaluate. However, it appears that antimetabolites are at high risk of such inter species target cell differences and are probably not suitable for pharmacologically guided dose escalation.. Drugs that exhibit non-linear kinetics provide further problems. For such agents the AUC may rise rapidly with small increments in dose providing a risk of inadvertent excessive toxicity.

In the phase I trial of amphethinile reported above the maximum dose was reached after four escalations. If the modified Fibonacci series had been used the same maximum dose would have taken 9–10 escalations requiring an additional 9–12 months work to complete the study. The use of pharmacological data to guide dose escalation was thus successful in eliminating the early stages of the trial, reducing the numbers of patients treated at low doses and significantly shortening the duration of the study. In addition the trial showed that a degree of non-linearity in the kinetics is not a bar to their use to guide dose escalation if care is taken.

Unfortunately two deaths occurred at the maximum dose used in the study. These deaths appeared to result from vascular causes and in both cases occurred in older patients who are at risk of pre-existing degenerative vascular disease. In neither case was systemic drug exposure high (AUC $<200\,\mu g\,l^{-1}\,h^{-1}$) although it is possible that some combined effect of the amphethinile+solutol was responsible for the effects. This demonstrates another problem with dose escalation based on pharmacological data. Such data cannot predict when toxic effects will occur that are not related to AUC and argues for caution in the later stages of trials. However highly unpredictable effects cannot be forseen by any method and are one of the inherent risks of phase I studies.

A further difficulty with this trial was that the formulation vehicle, solutol, had not been previously used in man. This raises the question of when such agents should be tested in man. Most patients enter phase I trials because they believe there is a chance that the treatment may help them. Would they be prepared to receive doses of a vehicle that had no likelihood of any effect on their disease? Logically solubilising agents should be tested prior to their inclusion in a formulation in order to avoid waste of resources if the agent proved to have unwanted biological effects. For solutol there was good scientific and animal data to suggest that it was considerably safer than the related Cremophor EL and thus a compromise design was used in this study where the solutol alone and the formulated drug were tested concurrently.

We believe that this trial demonstrates the potential for pharmacologically based dose escalation schemes in reducing the time and numbers of patients required for phase I trials. In addition a higher proportion of patients may be treated at potentially therapeutic doses. The trial also underlines the care that must be taken at higher doses irrespective of the dose escalation method in use.

Due to the occurrence of unpredictable neurological and vascular toxicities it was not possible to administer a dose of amphethinile that could achieve consistent cytotoxic effects in terms of neutropenia and alopecia. For this reason the drug cannot be recommended for phase II testing by the i.v. route. However amphethinile remains an interesting compound and future trials are planned using an oral formulation.

This work was supported by the Cancer Research Campaign.

### References

COLLINS, J.M., ZAHARKO, D.S., DEDRICK, R.L. & CHABNER, B.A. (1986). Potential roles for preclinical pharmacology in phase I clinical trials. *Cancer Treat. Rep.*, **70**, 73.

ESTEY, E.., HOTH, D., SIMON, R., MARSONI, S., LEYLAND-JONES, B. & WITTES, R. (1986). Therapeutic response in phase I trials of antineoplastic agents. *Cancer Treat. Rep.*, **70**, 1105.

MILLER, A.B., HOOGSTRATEN, B., STAQUET, M. & WINKLER, A. (1981). Reporting results of cancer treatment. *Cancer*, **47**, 201.